# *Lactobacillus johnsonii* LJO02 (DSM 33828) Cell-Free Supernatant and Vitamin D Improve Wound Healing and Reduce Interleukin-6 Production in *Staphylococcus aureus*-Infected Human Keratinocytes

**DOI:** 10.3390/pharmaceutics16010018

**Published:** 2023-12-21

**Authors:** Paola Zanetta, Chiara Ballacchino, Diletta Francesca Squarzanti, Angela Amoruso, Marco Pane, Barbara Azzimonti

**Affiliations:** 1 Laboratory of Applied Microbiology, Department of Health Sciences (DISS), Center for Translational Research on Allergic and Autoimmune Diseases (CAAD), School of Medicine, Università del Piemonte Orientale (UPO), Corso Trieste 15/A, 28100 Novara, Italy; 20028195@studenti.uniupo.it (C.B.); diletta.squarzanti@med.uniupo.it (D.F.S.); 2 Probiotical Research S.r.l., Via Mattei 3, 28100 Novara, Italy; a.amoruso@probiotical.com (A.A.); m.pane@probiotical.com (M.P.)

**Keywords:** *Staphylococcus aureus*, *Lactobacillus johnsonii*, vitamin D, multi-drug resistance, cell viability, wound healing, skin infection, skin inflammation, probiotic cell-free supernatant, skin cancer

## Abstract

Methicillin-resistant biofilm-forming *Staphylococcus* spp. are found in about 25% of the overall cases of chronic wounds, which can undergo malignant degeneration and be associated with skin cancer. Although antimicrobial agents are clinically used to counteract pathogens and promote wound healing, they are increasingly ineffective against multi-drug resistant bacteria. Moreover, they can induce dysbiosis, which favors opportunistic pathogen infections and alters immune responses. Consequently, research on pathogen containment strategies is crucial. We aimed to evaluate the potential beneficial effect of *Lactobacillus johnsonii* LJO02 cell-free supernatant (CFS) and vitamin D, as single treatments or in combination, on cell viability, wound healing, and the pro-inflammatory interleukin-6 (IL-6) production of a *Staphylococcus aureus*-infected human immortalized keratinocyte cell line (HaCaT) in vitro model. The analysis showed that LJO02 CFS 20% *v*/*v* ratio and 100 nM vitamin D promoted infected cell viability and wound healing and significantly reduced IL-6 production. However, their effect was not synergic, since no significant difference between the single and combined treatments was observed. LJO02 CFS topic application and vitamin D supplementation could provide a valuable strategy for attenuating *S. aureus*-induced pathogenesis, promoting wound healing and opening new therapeutic strategies supporting the conventional approaches.

## 1. Introduction

In recent years, the link between skin dysbiosis, altered immune response, and skin disease development has been deeply investigated [1]. Particularly, skin dysbiosis may lead to both exogenous and endogenous *Staphylococcus aureus* infections, which have a pivotal role in the onset and progression of various local skin diseases, such as atopic dermatitis, psoriasis, acne vulgaris, and even chronic wounds. Notably, the latter has a demonstrated association with malignant degeneration, which can lead to skin cancer. Additionally, these conditions can have a systemic impact, adversely affecting human health, life quality, life expectancy, and resulting in significant sanitary costs [2,3,4,5,6]. Methicillin-resistant *Staphylococcus* spp. have been isolated in about 25% of the overall cases of chronic wounds [7,8]. Specifically, *S. aureus* represents the most common agent in chronic wounds, often occurring as a biofilm-forming bacterium resistant to antimicrobial therapy [9]. It is a human opportunistic pathogen belonging to the ESKAPE group (*Enterococcus faecium*, *Staphylococcus aureus*, *Klebsiella pneumoniae*, *Acinetobacter baumannii*, *Pseudomonas aeruginosa*, and *Enterobacter* spp.) [10,11]. *S. aureus* is usually detected in the upper layer of wounds, and it has been shown to be mainly organized in biofilm, a protective matrix of extracellular polymeric substances that confers resistance to traditional antibiotics and host immune defenses. In particular, within this complex structure, different microbial genera, species, and phyla aggregate and give rise to communities that, by exchanging information through the quorum sensing mechanism, can interact with each other and increase their pathogenicity potential [12].

Skin wound healing is an efficient and rapid natural physiological reaction to tissue injury [13]. This multifaceted biological process involves many cell types, as well as extracellular and intracellular macromolecules. It consists of four highly integrated phases: hemostasis, inflammation, proliferation, and tissue remodeling or resolution. For successful wound healing, these four phases must occur in the correct sequence and within the appropriate timeframe. Interruptions, aberrancies, or prolongation of the process may lead to delayed wound healing or non-healing chronic wounds [14,15]. Non-healing wounds affect about 3 to 6 million people in the United States, with people older than 65 years accounting for 85% of the total events. The economic evaluation of chronic wounds suggests that their care and treatment are time consuming and cost billions of dollars every year [14,15].

Currently, antimicrobial agents are clinically used to counteract pathogens and promote wound healing [16]. However, their effectiveness is diminishing against multi-drug resistant (MDR) bacteria, inducing further dysbiosis and predisposing to opportunistic infections and immunity alterations [17,18,19,20]. Therefore, new methods to prevent, weaken, or break biofilm and limit the over-colonization and virulence of opportunistic pathogen biotypes are urgently needed [21]. 

Recently, lactic acid bacteria (LAB) and cell-free supernatants (CFSs) derived from their metabolisms have been investigated for their ability to reduce the virulence of different pathogenic species, including methicillin (oxacillin)-resistant *S. aureus* (MRSA) [22,23,24]. Furthermore, the literature reports strain specific *L. johnsonii* containment activity toward opportunistic pathogens such as *H. pylori* and *P. aeruginosa* [25,26]. Since we demonstrated that the CFS of the LJO02 strain (DSM 33828), isolated from the human gut of a healthy donor, inhibits MRSA viability and biofilm formation, in the present research, we examined its possible effect as a single agent or in combination with vitamin D on cell viability, wound healing, and pro-inflammatory interleukin-6 (IL-6) production on an MRSA-infected wounded human epithelial model. In particular, Squarzanti et al. demonstrated the potential of *Lactobacillus johnsonii* LJO02 CFS to inhibit MRSA viability and biofilm formation [2]. It has also been demonstrated that vitamin D can reduce the risk of infection through multiple mechanisms, such as boosting innate immunity via the modulation of anti-microbial peptides (AMPs) and pro-inflammatory cytokine production [27]. In addition, preclinical and clinical studies strongly suggest that vitamin D exerts an influence on host immunity, infectious diseases, and autoimmune conditions [28,29]. Clinical data have demonstrated that vitamin D deficiency is involved in many pathological processes, including several viral, bacterial and fungal infections and also chronicity of skin wounds [30,31].

On these premises, our aim was to search for novel alternatives to antibiotics and assess their effectiveness in the prevention and control of non-healed skin lesions infected with *S. aureus*, one of the main bacterial strains responsible for the global MDR emergency. Therefore, we examined the effect of LJO02 CFS as a single agent or in combination with vitamin D on cell viability, wound healing, and the pro-inflammatory interleukin-6 (IL-6) production on an MRSA-infected human immortalized keratinocyte (HaCaT) in vitro model.

Firstly, we assessed the growth behavior and biofilm formation ability of *S. aureus* and LJO02 in the presence of vitamin D in both their conventional and eukaryotic cell media. *S. aureus* was capable of adapting, growing, and forming biofilm in this latter medium, without being significantly influenced by the tested vitamin D concentrations. In contrast, LJO02 did not display a similar adaptability. Thus, we directly infected HaCaT cells with this pathogen while testing the ability of vitamin D and LJO02 CFS to counteract infection outcomes. As demonstrated by Squarzanti et al., this probiotic CFS reduced viable and metabolically active *S. aureus*, mainly through its organic acids, fatty acids, and proteins [2]. For the first time to our knowledge, we showed that LJO02 CFS or vitamin D applications can be a good strategy for attenuating *S. aureus*-induced pathogenesis, promoting wound healing and inflammation reduction, and thus opening new therapeutic frontiers supporting the conventional approaches.

## 2. Materials and Methods

### 2.1. Bacterial Cultures

*Staphylococcus aureus* (American Type Culture Collection, ATCC 43300, distributed by LGC Standards S.r.l., Sesto San Giovanni, Milan, Italy) was aerobically cultivated overnight (ON) at 37 °C and 200 rpm in Luria–Bertani broth (LB, Sigma-Aldrich, St. Louis, MO, USA, distributed by Merck Life Science S.r.l., Milan, Italy). The probiotic strain *Lactobacillus johnsonii* LJO02 (DSM 33828, kindly provided by Probiotical Research S.r.l., Novara, Italy), isolated in Italy from the human gut of a healthy donor, was aerobically cultivated ON at 37 °C in static conditions, using De Man, Rogosa and Sharpe broth (MRS, Condalab, distributed by Cabru S.A.S., Biassono, Italy). All bacterial strains were freshly renewed before each experiment.

### 2.2. Eukaryotic Cell Culture

A spontaneously immortalized human epidermal keratinocyte cell line (HaCaT; CLS Cell Lines Service GmbH, Eppelheim, Germany) was maintained in Dulbecco’s Modified Eagle’s Medium (DMEM; Cytiva, Logan, Utah, United States, distributed by CliniSciences S.r.l., Guidonia Montecelio, Rome, Italy) with L-glutamine (4 mM) and a high glucose concentration (4500 mg/L), without sodium pyruvate, and supplemented with 10% heat-inactivated fetal bovine serum (FBS; Corning, Glendale, AZ, USA, distributed by Biosigma S.p.A., Cona, Venice, Italy) and 1% penicillin and streptomycin mixture (10,000 units/mL penicillin and 10 mg/mL streptomycin mixture, Sigma-Aldrich). The cells were grown in a humidified 5% CO_2_ atmosphere at 37 °C. For all the experiments, HaCaT cells were seeded into 48-well plates (2 × 10^5^ cells/mL, 500 μL/well) or 96-well plates (1 × 10^5^ cells/mL, 100 μL/well) in a complete growth medium without antibiotics. The cells were PCR-tested for mycoplasma contamination every four weeks.

### 2.3. Bacterial Growth Curves

To figure out the initial optical density (OD) needed in the experimental conditions, the *S. aureus* ON culture was diluted to an OD at 600 nm (OD_600_) of 0.01 and incubated at 37 °C as described above. The OD_600_ of 1 mL *S. aureus* suspension was measured every hour for 8 h using the NanoPhotometer NP80 (Implen, Munich, Germany). To determine the optimal endpoint for LJO02 CFS preparation, the ON probiotic culture was diluted to an OD_600_ of 0.05 and incubated at 37 °C in optimal growth conditions. The OD_600_ was measured every 2 h until 8 h and at 24, 48, and 72 h, as described for the *S. aureus* growth curve. To evaluate their capability to also adapt and grow in eukaryotic cell culture conditions, growth curves for *S. aureus* and LJO02 were assessed both in their conventional and in DMEM 10% FBS media. In addition, the experiment was also performed in the presence of vitamin D (1α,25-dihydroxyvitamin D3, Cabru S.A.S.) to assess whether this supplement could affect bacterial growth. In this case, fresh *S. aureus* and LJO02 cultures were diluted in their conventional media at an OD_600_ of 0.035 and 0.05, respectively, plated into 48-well plates, and immediately treated with different concentrations of vitamin D (1 μM, 100 nM, 10 nM, and 1 nM) in dimethyl sulfoxide (DMSO; Merck Life Science S.r.l.). For the growth curves in DMEM 10% FBS, fresh cultures were centrifuged at 4000 rpm (Heraeus Megafuge 16R, Thermo Fisher Scientific, Rodano, Milan, Italy) for 15 min at room temperature (RT), resuspended in the complete eukaryotic cell medium without antibiotics, and plated and measured as described above. In all the experiments, DMSO and DMEM 10% FBS without antibiotics were used as controls. At each timepoint, the OD_600_ was read using a Spark microplate reader (Tecan Italia S.r.l, Milan, Italy). Each experiment was conducted with four replicates per condition and repeated three times independently.

### 2.4. Crystal Violet Biofilm Staining

To assess the biofilm formation ability in the two different media, crystal violet (CV) staining of the biofilm produced by *S. aureus* and LJO02 cultivated in their conventional media and in DMEM 10% FBS with different concentrations of vitamin D was performed. *S. aureus* and LJO02 were respectively plated at an OD_600_ of 0.035 and 0.05 into 48-well plates and immediately treated with vitamin D, as described above. A plate for each bacterium and endpoint of 24, 48, and 72 h was used. At the defined endpoints, the supernatant was removed, and the biofilm was fixed with pure methanol (Sigma-Aldrich) for 15 min at RT. Then, the dried biofilm was stained with 1% CV solution (Sigma-Aldrich) for 5 min at RT. After removing the CV excess, the biofilm images were acquired with EVOS FLoid^TM^ Cell Imaging Station (Thermo Fisher Scientific). To quantify the biofilm amount, a 33% acetic acid solution (Sigma-Aldrich) was used to dissolve the CV and the absorbance was read at 570 nm with a Spark microplate reader. Each experiment was conducted with four replicates per condition and repeated three times independently.

### 2.5. Cell-Free Supernatant (CFS) Production

A fresh culture of LJO02 was inoculated at OD_600_ = 0.05 into MRS broth and incubated for 8 h in proper conditions. Then, the bacterial culture was centrifuged at 4000 rpm for 20 min at 4 °C. After bringing the pH to 7 with NaOH 5N solution, the CFS was sterilized with a 0.22 μm polyethersulfone (PES) filter (Clearline, distributed by Biosigma), aliquoted, and stored at −20 °C until use. Pristine MRS culture medium was incubated, centrifuged, filtered, and stored as the CFS and used as the control in the following experiments (iMRS). The protein content and lactic acid amount were quantified with the bicinchoninic acid (BCA) Protein Assay Kit (Biosciences, St. Louis, MO, USA, distributed by Cabru S.A.S.) and the D/L-Lactic Acid Megazyme Assay Kit (NEOGEN Europe Ltd., Ayr, UK), following the manufacturer’s instructions.

### 2.6. Viability Assay

The CellTiter-Glo^®^ Luminescent Cell Viability Assay (Promega, Italia S.r.l., Milan, Italy) was performed following the manufacturer’s instructions on HaCaT cells treated with different LJO02 CFS *v*/*v* ratios (50, 40, 30, 20, 10, and 5%) or vitamin D concentrations (1 μM, 100 nM, 10 nM, and 1 nM) to select non-toxic conditions for this cell line. Moreover, a viability assay was conducted on infected HaCaT cells with *S. aureus* at 100 MOI, treated with LJO02 CFS (20, 10, and 5%) and/or vitamin D 100 nM for 24 h to assess whether the treatments could preserve cell viability upon pathogen infection. Each experiment was performed in triplicate and repeated three times independently.

### 2.7. Wound Healing Assay

HaCaT cells were seeded into a 48-well plate and incubated for 24 h, allowing the cells to form a confluent monolayer. Then, the medium was carefully removed, the wells were washed with PBS 1×, and 200 µL tips were used to make the wounds. Finally, the cells were treated with vitamin D at different concentrations (1 μM, 100 nM, 10 nM, and 1 nM) or with LJO02 CFS (20, 10, and 5% *v*/*v*). DMEM 2.5% FBS was used to reach the final volume of 500 μL/well. DMSO, iMRS, and DMEM 2.5% FBS were used as controls. Moreover, the same experiment was repeated in the presence of *S. aureus* infection at 100 MOI to assess the ability of LJO02 CFS (20% *v*/*v*) and/or vitamin D (100 nM) to improve wound healing in case of an infected wound. To analyze cell migration, representative images focused on the wound field were photographed at time 0 and after 24 h, using the Leica software application suite (LAS EZ 2.1.0) connected to an optical microscopy at 4× magnification (Leica ICC50 HD). The pictures were analyzed using the open-source ImageJ software v1.53s (Rasband, W.S., ImageJ, U. S. National Institutes of Health, Bethesda, MD, USA, https://imagej.nih.gov/ij/ (accessed on 16 June 2022), 1997–2018). The wound area reduction was calculated for each set and plotted on a graph with standard deviation (SD). The wound healing percentage at 24 h was calculated using the following formula:

$$Wound\;healing\;\%=\frac{Open\;area\;T_{0}-Open\;area\;T_{24}}{Open\;area\;T_{0}}\times 100$$

Each experiment was conducted with four replicates per condition and repeated three times independently.

### 2.8. IL-6 ELISA Assay

For the quantitative detection of IL-6 released from HaCaT cells after *S. aureus* infection and/or treated with LJO02 CFS and/or with vitamin D, a commercial human IL-6 ELISA Kit (FineTest ^®^, Wuhan, China, distributed by Cabru S.A.S.) was used. Briefly, HaCaT cells were seeded into 96-well plates and infected and/or treated as described above and incubated for 4 h. Then, the supernatants were collected and centrifuged for 5 min at 2500 rpm at 4 °C to remove insoluble impurities and cell debris. The supernatant was then used for IL-6 quantification following the manufacturer’s instructions.

### 2.9. Statistical Analysis

One-way and two-way ANOVA followed by Tukey multiple comparisons were performed using the GraphPad Prism version 7.04 for Windows (GraphPad Software, San Diego, CA, USA, www.graphpad.com (accessed on 15 June 2018)). The results were expressed as mean ± standard deviation (SD). The statistical significance was fixed at *p* < 0.05.

## 3. Results and Discussion

### 3.1. Bacterial Growth Curves

**Figure 1:**
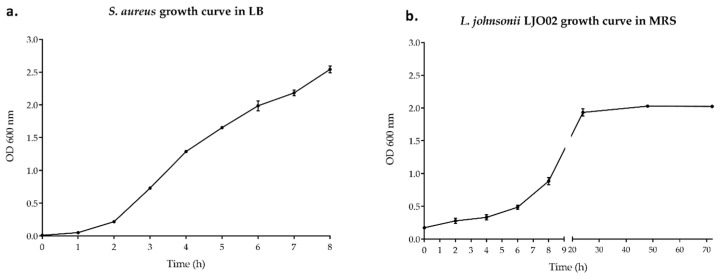
Growth curves of (**a**) *S. aureus* in LB and (**b**) LJO02 in MRS. Data are expressed as the mean of three independent experiments ± SD. OD 600 nm = optical density at 600 nm.

*S. aureus* and LJO02 growth curves were obtained in the standard LB and MRS media, respectively (Figure 1). Based on the results obtained, we determined that OD_600_ = 0.035 corresponded to the beginning of the *S. aureus* exponential phase. Therefore, the pathogen was diluted to reach this OD value to assess its growth in the presence of vitamin D in LB or in DMEM 10% FBS (Figure 1a). LJO02 reached the end of its exponential phase after 8 h of incubation, and thus this endpoint was selected to produce its CFS (Figure 1b).

**Figure 2:**
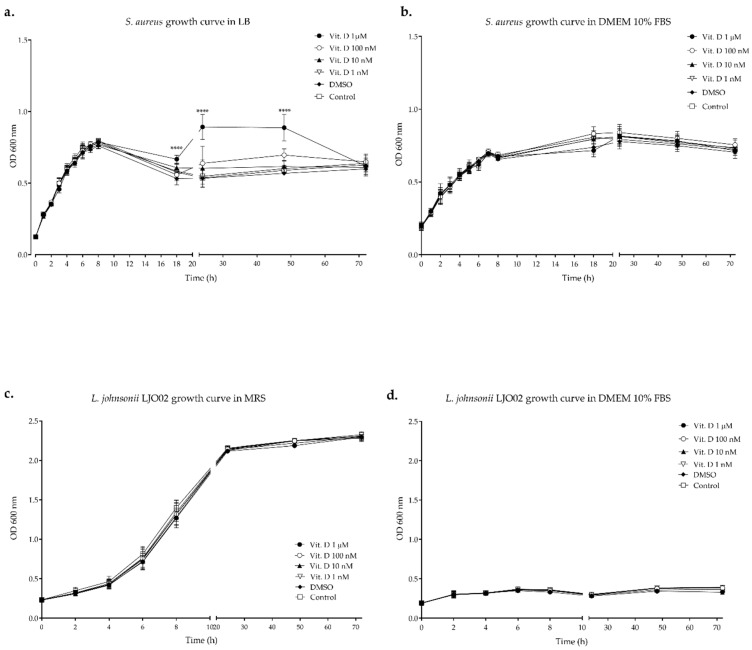
*S. aureus* and LJO02 growth curves in their standard media and DMEM 10% FBS with vitamin D. (**a**,**b**) *S. aureus* growth curves in LB and DMEM 10% FBS with different vitamin D concentrations; (**c**,**d**) LJO02 growth curves in MRS and DMEM 10% FBS with different vitamin D concentrations. Data are expressed as the mean of three independent experiments ± SD. OD 600 nm = optical density at 600 nm. **** *p* < 0.0001.

The growth curves of *S. aureus* and LJO02 in their elective growth media and in DMEM 10% FBS with different vitamin D concentrations are shown in Figure 2. *S. aureus* growth was significantly higher in LB compared to DMEM 10% FBS between 5 and 8 h of incubation (*p* < 0.0001 at 5, 6, and 8 h; Figure 2a,b). However, the pathogen adapted well to DMEM 10% FBS, and its growth rate even increased in DMEM 10% FBS compared to LB starting from 11 h, with a significant difference after 18 h of incubation (*p* < 0.0001 at 18, 24, 48, and 72 h; Figure 2a,b). The growth curves of *S. aureus* in both media supplemented with vitamin D showed that this compound had no effect on bacterial growth in all the conditions, except for LB supplemented with vitamin D 1 μM, compared to other vitamin D concentrations and controls (*p* < 0.0001 at 18, 24, and 48 h; Figure 2a,b). The growth curve of LJO02 in DMEM 10% FBS showed that the probiotic did not adapt to this medium (*p* < 0.0001 at 4, 6, 8, 24, 48, and 72 h vs. MRS; Figure 2c,d). The presence of vitamin D did not influence the growth of LJO02 in both media (Figure 2c,d). In conclusion, the growth curves showed that *S. aureus* adapted and duplicated in eukaryotic cell culture conditions, giving the possibility to directly infect HaCaT cells. Moreover, its growth in DMEM 10% FBS was not influenced by vitamin D. Conversely, LJO02 did not adapt and duplicate in eukaryotic cell culture conditions independently from vitamin D, and thus we decided to assess the effects of its CFS.

### 3.2. Bacterial Biofilm Formation

**Figure 3:**
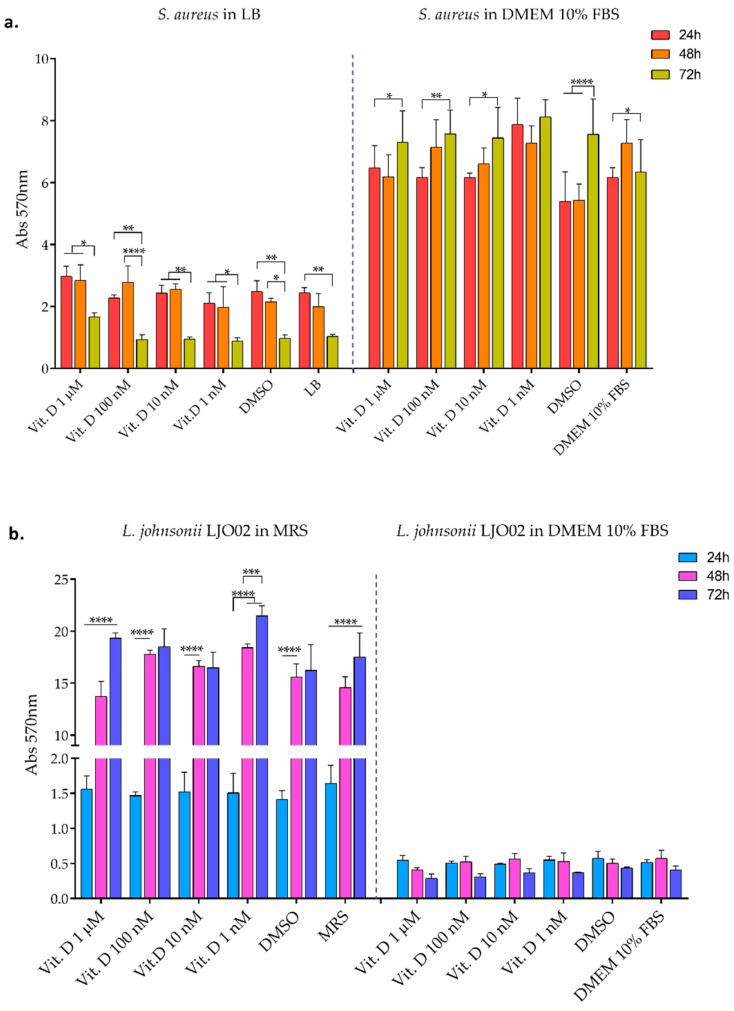
Biofilm quantification with crystal violet (CV) staining. (**a**) *S. aureus* and (**b**) LJO02 were assayed to determine their ability to form biofilm in their respective conventional culture media and in DMEM 10% FBS, both supplemented with different vitamin D concentrations. Data are represented as the mean of three independent experiments ± SD. Abs 570 nm = absorbance at 570 nm. * *p* < 0.05, ** *p* < 0.01, *** *p* < 0.001, **** *p* < 0.0001.

**Figure 4:**
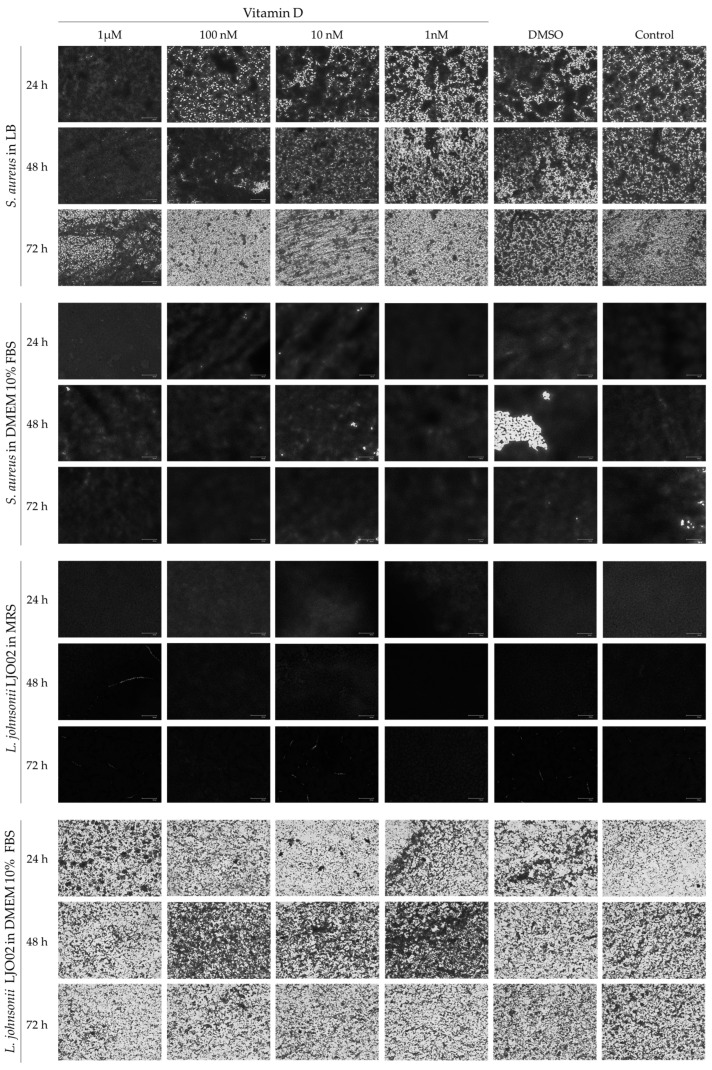
Representative crystal violet-stained biofilm images. Images were obtained with FLoid^TM^ Cell Imaging Station. Magnification 460×.

The formation of *S. aureus* and LJO02 biofilms, when cultivated in their elective media and in DMEM 10% FBS with different vitamin D concentrations, was also determined through CV staining at 24, 48, and 72 h. *S. aureus* adhered and grew as biofilm in all conditions (Figure 3a). In particular, a decreased biofilm amount was observed over time when it was cultured in LB, and vice versa, the biofilm increased over time in the presence of DMEM 10% FBS, as shown by the statistic reported in the graph (Figure 3a). LJO02 only maintained the capacity to form biofilm in MRS (Figure 3b), where its amount significantly increased over time. However, the behavior of both bacteria seemed not to be influenced by the presence of vitamin D. Figure 4 shows representative images of CV-stained biofilm which support the absorbance results. After proving that vitamin D did not inhibit per se pathogen growth and virulence, we wanted to assess whether its combination with LJO02 CFS could improve the containment of *S. aureus* effects on infected HaCaT cells. Moreover, we faced again the need for a probiotic CFS use, instead of the viable strain, as it has already shown its ability to inhibit *S. aureus* growth and biofilm formation [2].

### 3.3. L. johnsonii LJO02 CFS Analysis

Mass spectrometry analysis of LJO02 CFS produced in MRS had already been conducted by our group [2]. However, to assess the reproducibility of the CFS production, the protein and lactic acid contents were analyzed in addition to recording the pH value. In Table 1, the results obtained from this analysis are reported. LJO02 CFS was used at pH 7 on HaCaT cells, as described in the materials and methods.

### 3.4. LJO02 CFS and Vitamin D Effects on HaCaT Cells Viability

**Figure 5:**
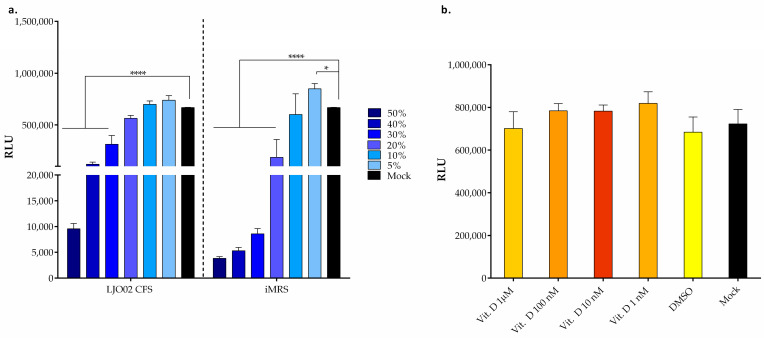
Effect of LJO02 CFS and vitamin D on HaCaT cells viability at 24 h. (**a**) LJO02 CFS was used at different *v*/*v* ratios of 50, 40, 30, 20, 10, and 5%; (**b**) vitamin D was used at the concentrations of 1 μM, and 100, 10, and 1 nM. The graph represents means ± SD of three independent experiments, each performed in triplicate. * *p* < 0.05; **** *p* < 0.0001. RLU = relative luminescence unit.

To determine the best concentration range of LJO02 CFS and vitamin D, a viability assay at 24 h was performed using different concentrations of these substances (Figure 5). A dose-dependent decrease in HaCaT cell viability was observed by increasing the concentration of LJO02 CFS. In particular, the resulting cell viability significantly reduced when treated with LJO02 CFS only at 50, 40, and 30% when compared to the untreated control (mock, *p* < 0.0001; Figure 5a). Conversely, the LJO02 CFS at 20 and 30% significantly increased HaCaT cell viability in comparison to the relative controls in iMRS. The conditions iMRS at 50, 40, 30, and 20% were associated with a significant reduction in cell viability compared with the mock (*p* < 0.0001; Figure 5a). The iMRS 10% did not significantly affect the cellular viability. Instead, iMRS 5% was associated with an increased cell viability compared to the mock (*p* < 0.05; Figure 5a). When different concentrations of vitamin D were used, no statistically significant differences were observed in HaCaT cell viability and DMSO control compared to the mock (Figure 5b). Thus, vitamin D did not affect HaCaT cell viability. Based on these results, LJO02 CFS concentrations at 20, 10, and 5% were selected for the next experiments, while all vitamin D concentrations were further investigated on HaCaT cell wound healing. 

### 3.5. LJO02 CFS and Vitamin D Effects on HaCaT Cells Wound Healing

**Figure 6:**
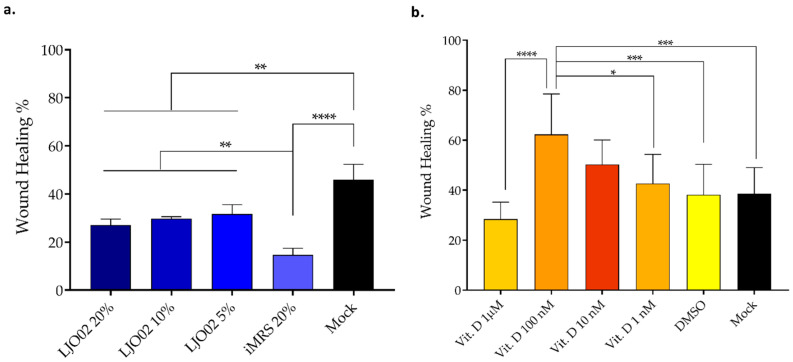
Percentage of HaCaT cell wound healing at 24 h. (**a**) LJO02 CFS effect on HaCaT cell wound healing; (**b**) vitamin D effect on HaCaT cell wound healing. The averages of the opened wound area were measured with the ImageJ software and plotted as a relative percentage of the original wound. The bar graph represents the means ± SD of three independent experiments, each performed in quadruplicate. * *p* < 0.05; ** *p* < 0.01; *** *p* < 0.001; **** *p* < 0.0001.

**Figure 7:**
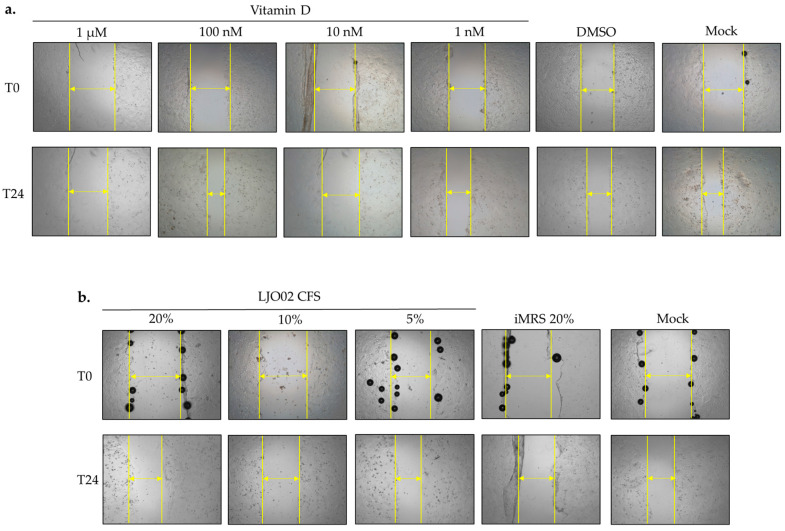
Representative images of HaCaT cells wound healing at 0 and 24 h. (**a**) LJO02 CFS effect on HaCaT cell wound healing; (**b**) vitamin D effect on HaCaT cell wound healing. Yellow arrows indicate the distances used to calculate the percentage of wound healing. Magnification 4×.

To test the hypothesis that vitamin D and LJO02 CFS can promote keratinocyte proliferation following an induced scratch, a wound healing assay was performed (Figure 6). Firstly, the selected LJO02 CFS *v*/*v* ratios (20, 10, and 5%) were assessed (Figure 6a). While all conditions were associated with a significantly reduced scratch re-epithelization compared to the mock (*p* < 0.01; Figure 6a), no significant differences were observed among the tested CFS concentrations. In contrast, they were associated with a statistically significant improvement in HaCaT cells wound healing compared to the iMRS 20% treated cells (*p* < 0.01; Figure 6a). Regarding vitamin D, only the 100 nM concentration significantly increased the re-epithelialization of the HaCaT cell monolayer compared to vitamin D 1 μM (*p* < 0.0001), DMSO (*p* < 0.001), and the mock (*p* < 0.001; Figure 6b). Vitamin D 100 nM re-epithelized 63.30% of the scratched area. Conversely, vitamin D 1 μM, DMSO, and the mock control resulted only in 28.38%, 38.14%, and 38.51% of re-epithelization, respectively. Vitamin D 10 and 1 nM did not significantly stimulate monolayer re-epithelization compared to DMSO and the untreated control (Figure 6b). Thus, vitamin D 100 nM was selected as the optimal concentration to promote HaCaT cell wound healing, and its role was further evaluated in *S. aureus*-infected HaCaT cells. In Figure 7, representative images of the wound healing experiments are shown.

This result reinforces the data on the efficacy of vitamin D in promoting wound healing demonstrated by other authors [32,33]. Guidelines from different scientific societies and countries defined the physiological range of vitamin D in the blood as 30–100 ng/mL. Considering that vitamin D 100 nM is equivalent to 38.4 ng/mL, this concentration is within the physiological range [33]. According to Bikle et al., the physiological concentration of vitamin D promotes in vitro wound healing, whereas vitamin D deficiency results in delayed scratch re-epithelialization or chronic wounds, suggesting the usefulness of vitamin D supplementation only in a deficiency context [34].

### 3.6. LJO02 CFS and Vitamin D Effects on S. aureus-Infected HaCaT Cell Viability

**Figure 8:**
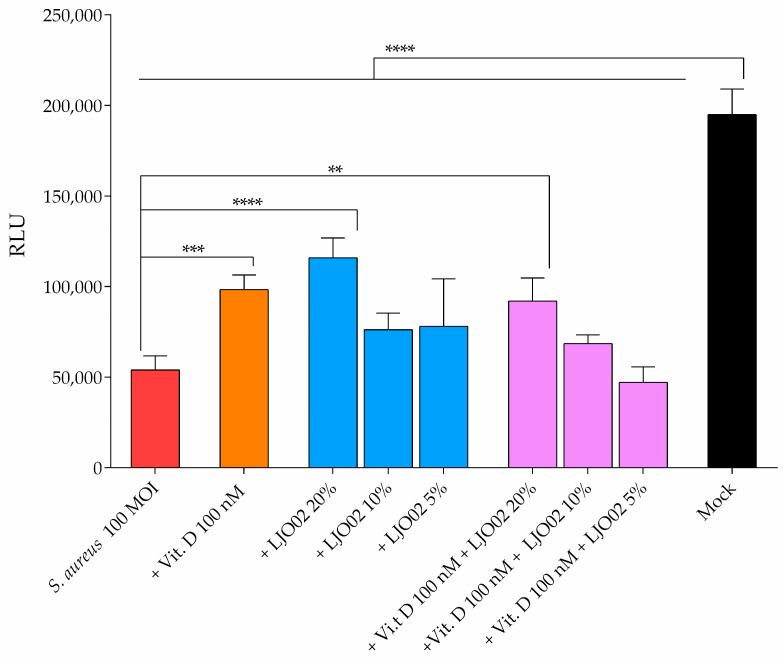
Effect of LJO02 CFS (20, 10, and 5%) and/or vitamin D 100 nM on *S. aureus* (100 MOI)-infected HaCaT cell viability at 24 h. The graph represents the means ± SD of three independent experiments, each one performed in triplicate. ** *p* < 0.01; *** *p* < 0.001; **** *p* < 0.0001. RLU = relative luminescence unit.

HaCaT cells were infected with *S. aureus* at 100 MOI, since this has been identified as the ideal concentration for a slow kinetic of infection, mimicking the in vivo scenario in which this bacterium turns into a pathogenic phenotype [8,35]. To determine the potential protective effect of vitamin D and LJO02 CFS on HaCaT cell viability, infected cells were treated with vitamin D 100 nM and/or LJO02 CFS at 20, 10, and 5% for 24 h (Figure 8). The *S. aureus* infection resulted in a statistically significant reduction in HaCaT cell viability compared to the untreated and uninfected control (mock, *p* < 0.0001; Figure 8). Vitamin D 100 nM significantly increased the viability of infected cells in comparison to untreated cells (*p* < 0.001; Figure 8). No statistically significant improvement in HaCaT cell viability was observed in infected cells treated with the LJO02 CFS at 5 and 10%. Conversely, LJO02 CFS at 20% significantly increased the viability of infected cells (*p* < 0.0001; Figure 8). Therefore, based on this result and considering that we previously observed the containment of *S. aureus* growth with a 50% *v*/*v* ratio [2], LJO02 CFS at 20% was selected to investigate its capability to contain the infection effects on HaCaT cells. The combined treatment of infected cells with vitamin D 100 nM and LJO02 CFS at different concentrations showed that only the combination of vitamin D and LJO02 CFS at 20% significantly increased HaCaT cell vitality compared to the mock (*p* < 0.01; Figure 8). Therefore, the LJO02 CFS at 20% was selected as the optimal concentration for further experiments, as it did not show any toxic effect on HaCaT cells, and it significantly attenuated the *S. aureus* infection outcome on cell viability. No statically significant difference was observed between the combined vitamin D 100 nM and LJO02 CFS at 20% treatment and the single ones. Despite the increased viability of treated HaCaT cells compared to the infected ones, the values were still significantly reduced when compared to the mock (*p* < 0.0001; Figure 8), suggesting that these treatments can mitigate the effect of *S. aureus* infection without restoring the viability values.

The effect of *S. aureus* on HaCaT cells is mainly attributed to its virulence factors. Among them, secreted toxins play a key role. Its main toxins are subdivided into pore-forming toxins (PFTs), exfoliative toxins (ETs), and superantigens (SAgs) [5,36]. An additional mechanism for the damage of infected keratinocytes with *S. aureus* is the adherence and subsequent invasion of the cells by the bacteria, and it is thought that because of their non-professional phagocytic nature, keratinocytes cannot effectively kill the internalized bacteria [8]. Interestingly, the treatment with LJO02 CFS 20% and vitamin D resulted in an attenuation of the *S. aureus* effect on cell viability. While the combinatorial treatment of LJO02 CFS and vitamin D is still effective, this does not suggest a synergistic effect of these substances. Moreover, it should be noted from the bacterial growth curves that vitamin D does not have a direct effect on *S. aureus* growth, even though it is associated with an improvement in the viability of *S. aureus*-infected HaCaT cells, suggesting its indirect role in counteracting *S. aureus*. This result is consistent with recent findings demonstrating that the active form of vitamin D regulates the transcription of AMPs, including LL-37, cathelicidin antimicrobial peptide (CAMP), and beta-defensin-2 in keratinocytes, macrophages, and neutrophils; thus, vitamin D is implicated in the host immune response to bacteria [27,30,37].

### 3.7. LJO02 CFS and Vitamin D Effects on S. aureus-Infected HaCaT Cell Wound Healing

**Figure 9:**
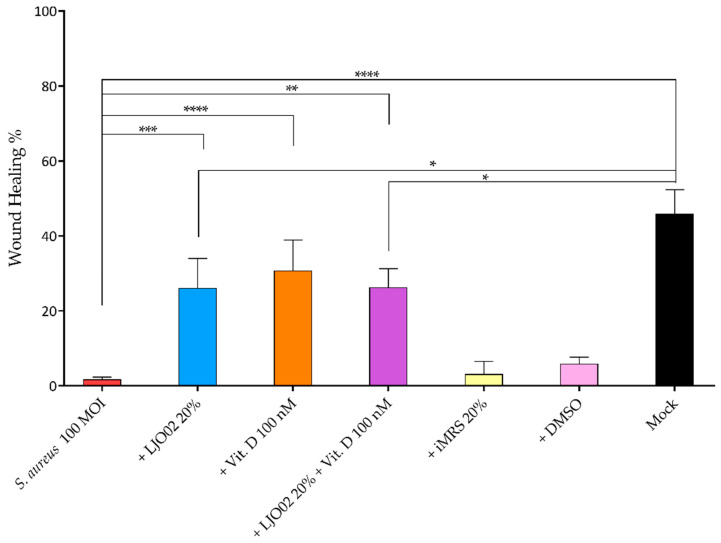
Effect of LJO02 CFS 20% and/or vitamin D 100 nM on the wound healing of *S. aureus*-infected HaCaT cells at 24 h. The averages of the opened wound area were measured with the ImageJ software and plotted as a relative percentage of the original wound. The graph represents the means ± SD of three independent experiments, each performed in quadruplicate. * *p* < 0.05; ** *p* < 0.01; *** *p* < 0.001; **** *p* < 0.0001.

**Figure 10:**
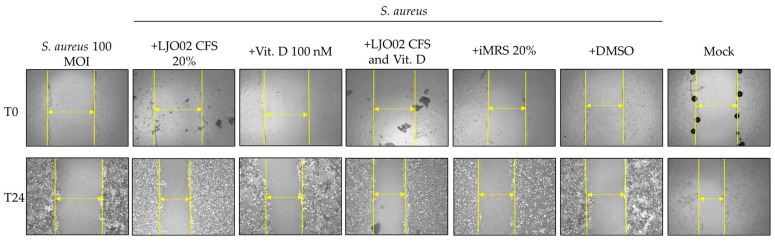
Representative images of the wound healing of *S. aureus*-infected HaCaT cells at 0 and 24 h. Yellow arrows indicate the distances used to calculate the percentage of wound healing. Magnification 4×.

To test the hypothesis whether vitamin D and LJO02 CFS can promote keratinocyte wound repair following an induced scratch in *S. aureus*-infected HaCaT cells, a wound healing assay was performed (Figure 9). The infection of HaCaT cells with *S. aureus* at 100 MOI resulted in a significant reduction in scratch re-epithelization (1.72%) compared to the mock (45.93%, *p* < 0.0001; Figure 9). A statistically significant acceleration of wound healing was observed for *S. aureus*-infected cells treated with LJO02 CFS 20% (26.10%, *p* < 0.001), vitamin D 100 nM (30.79%, *p* < 0.0001), and their combined treatment (26.31%, *p* < 0.01; Figure 9). No statistically significant difference was observed between these three conditions, thus suggesting the absence of a synergistic effect. The scratch closure area, however, was still significantly lower than the one for the mock for LJO02 CFS 20% and for its combined treatment with vitamin D 100 nM (*p* < 0.05; Figure 9). These results confirm that vitamin D and LJO02 CFS as single treatments have a positive effect on infected cell wound healing without reaching the same healing intensity as in the absence of infection. Figure 10 shows representative images of the wound healing experiments. 

The different pieces of evidence underline that the colonization of skin wounds with commensal bacteria may promote the healing process by inducing antimicrobial proteins, such as Perforin-2 (P-2), thus stimulating a protective immune response against pathogenic bacteria. Conversely, a wound infection with pathogenic bacteria results in P-2 suppression and healing inhibition. *S. aureus* significantly reduces HaCaT cell scratch closure through different mechanisms, such as the evasion of the antimicrobial activity of P-2 and biofilm formation [9,38]. Interestingly, the LJO02 CFS and vitamin D treatments of *S. aureus*-infected HaCaT cells are associated with a significant acceleration of scratch closure, while the combined treatment does not amplify the effect of the individual agents. This study was the first demonstrating the protective effect of LJO02 CFS and vitamin D on *S. aureus*-infected wound healing, although several studies have already demonstrated that different CFSs exert a positive effect on infected wounds [14,39].

### 3.8. LJO02 CFS and Vitamin D Effect on IL-6 Production by S. aureus-Infected HaCaT Cells

**Figure 11:**
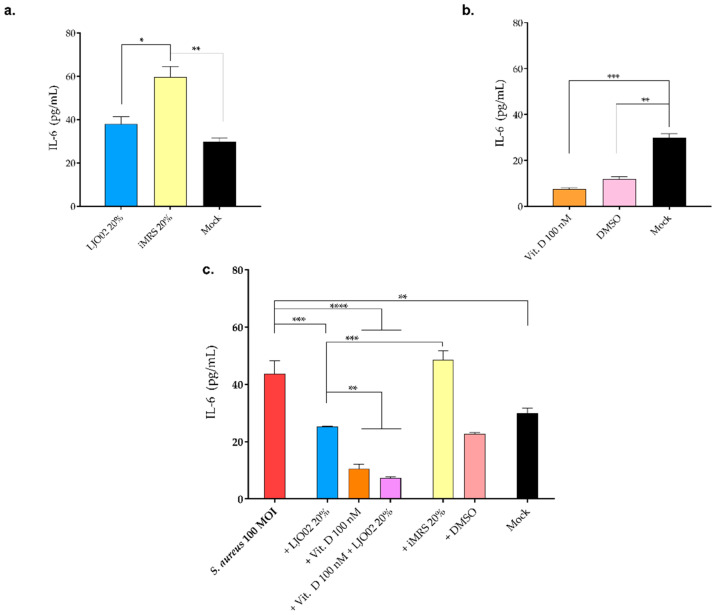
IL-6 response induced in HaCaT cells at 4 h. (**a**) LJO02 CFS 20% and (**b**) vitamin D 100 nM induced IL-6 production by HaCaT cells. (**c**) LJO02 CFS 20% and/or vitamin D 100 nM effect on IL-6 production by *S. aureus* (100 MOI)-infected HaCaT cells. The graph represents the means ± SD of three independent experiments, each performed in triplicate. * *p* < 0.05; ** *p* < 0.01; *** *p* < 0.001; **** *p* < 0.0001.

An ELISA assay was performed to assess the effect of the selected LJO02 CFS and vitamin D concentrations on HaCaT cell IL-6 production (Figure 11). HaCaT cells were treated with vitamin D 100 nM or the LJO02 CFS 20% for 4 h. As shown in Figure 11a, no significant differences in IL-6 values were assessed between the LJO02 CFS and the mock. On the other hand, iMRS 20% significantly increased IL-6 levels compared with both the LJO02 CFS (*p* < 0.05; Figure 11a) and the mock control (*p* < 0.01; Figure 11a), underlining the pro-inflammatory effect of iMRS, which is attenuated by the presence of the bacterial metabolic products present in the probiotic CFS. Vitamin D 100 nM and its solvent DMSO significantly reduced IL-6 production by HaCaT cells compared with the mock (*p* < 0.001 and *p* < 0.01; Figure 11b), whereas no statistically significant differences in IL-6 production were observed between vitamin D and DMSO (Figure 11b). When HaCaT cells were infected with *S. aureus* at 100 MOI, a statistically significant increase in IL-6 production compared to the mock was induced (*p* < 0.01; Figure 11c). The treatment of *S. aureus*-infected HaCaT cells with LJO02 CFS significantly reduced IL-6 levels compared to infected cells (*p* < 0.001; Figure 11c), restoring the IL-6 values observed in the mock. iMRS 20% was still associated with an increased IL-6 production in comparison with all the other investigated conditions. The supplementation of vitamin D 100 nM and the combined treatment with vitamin D and LJO02 CFS led to a significant reduction in IL-6 values compared to both infected cells (*p* < 0.0001; Figure 11c) and LJO02 CFS-treated cells (*p* < 0.001; Figure 11c). Therefore, the treatment of *S. aureus*-infected HaCaT cells with LJO02 CFS or vitamin D as single agents or in combination attenuated the pro-inflammatory response associated with the bacterial infection.

The supplementation of HaCaT cells with vitamin D 100 nM is associated with a negative modulation of IL-6 secretion. This result is consistent with several reports demonstrating the critical role of vitamin D in immune system modulation [28,31,40]. In particular, vitamin D downregulates the production of pro-inflammatory cytokines such as IL-6, Interferon-γ (IFN-γ), IL-2, and TNF-α, suggesting its immunomodulatory effect [29,41]. Several pieces of evidence have already demonstrated that *S. aureus* is associated with an increased IL-6 production by HaCaT cells [42,43]. According to Ngo et al., the secretion of IL-6 by HaCaT cells is proportional to the internalization rate of *S. aureus* [11]. Additionally, IL-6 might prevent *S. aureus* from spreading to still unaffected healthy host cells by promoting keratinocyte differentiation and, thus, accelerate the disposal of the already infected and surrounding tissue [11]. Interestingly, the treatment with LJO02 CFS and vitamin D attenuated the release of IL-6 by *S. aureus*-infected cells, suggesting the inflammatory immunomodulation activity of both LJO02 CFS and vitamin D. Although no evidence obtained using the same in vitro model is reported in the literature, the generalized anti-inflammatory effects of vitamin D and LAB CFSs during *S. aureus* infection in different in vitro and in vivo models have been demonstrated [44,45].

## 4. Conclusions

Further experiments will be useful to explore pathological host–microbiota interactions and the molecular mechanisms underlying the effects of LJO02 CFS and vitamin D on *S. aureus*-infected cells, skin wound healing, and inflammatory modulation. Moreover, the use of more complex in vitro models, such as the more sophisticated three-dimensional cultures with immunocompetent skin cells, will better allow for the reproduction of the in vivo conditions. Since the combined LJO02 CFS and vitamin D treatment did not exploit a synergistic effect, it may be worth assessing whether it is dependent from the timing of the treatment. However, the effect of single LJO02 and vitamin D treatments on infected cell viability, wound healing, and IL-6 reduction is undeniable. 

According to the literature data, vitamin D activity has been demonstrated in wound repair or toward pathogens such as *S. aureus.* For the first time to our knowledge, we showed, in an *S. aureus*-infected wounded epithelial model, that vitamin D (or LJO02 CFS) could represent a novel strategy for attenuating *S. aureus*-induced pathogenesis, promoting wound healing and reducing bacterium-mediated inflammation. Therefore, this approach will hopefully open new therapeutic frontiers supporting or substituting the conventional antibiotic ones which favor MDR.

Thus, the topic application of LJO02 CFS and vitamin D supplementation in deficient patients could be a good strategy to open new therapeutic frontiers to support the conventional and sometimes and somehow MDR-promoting approaches.

## Figures and Tables

**Table 1:** LJO02 CFS characterization.

Protein Concentration (mg/mL)	pH	Lactic Acid Concentration (g/L)
8.52	4	7.11

Data represent the preliminary characterization of LJO02 CFS produced in MRS medium.

## Data Availability

All available data are presented in this paper.

## References

[1] De Pessemier B., Grine L., Debaere M., Maes A., Paetzold B., Callewaert C. (2021). Gut–Skin Axis: Current Knowledge of the Interrelationship between Microbial Dysbiosis and Skin Conditions. Microorganisms.

[2] Squarzanti D.F., Zanetta P., Ormelli M., Manfredi M., Barberis E., Vanella V.V., Amoruso A., Pane M., Azzimonti B. (2022). An animal derivative-free medium enhances Lactobacillus johnsonii LJO02 supernatant selective efficacy against the methicillin (oxacillin)-resistant Staphylococcus aureus virulence through key-metabolites. Sci. Rep..

[3] Kullander J., Forslund O., Dillner J. (2009). Staphylococcus aureus and squamous cell carcinoma of the skin. Cancer Epidemiol. Biomark. Prev..

[4] Squarzanti D.F., Zavattaro E., Pizzimenti S., Amoruso A., Savoia P., Azzimonti B. (2020). Non-Melanoma Skin Cancer: News from microbiota research. Crit. Rev. Microbiol..

[5] Oliveira D., Borges A., Simões M. (2018). Staphylococcus aureus Toxins and Their Molecular Activity in Infectious Diseases. Toxins.

[6] Tong S.Y.C., Davis J.S., Eichenberger E., Holland T.L., Fowler V.G. (2015). Staphylococcus aureus infections: Epidemiology, pathophysiology, clinical manifestations, and management. Clin. Microbiol. Rev..

[7] Pinto D., Ciardiello T., Franzoni M., Pasini F., Giuliani G., Rinaldi F. (2021). Effect of commonly used cosmetic preservatives on skin resident microflora dynamics. Sci. Rep..

[8] Wiegand C., Abel M., Ruth P., Hipler U.-C. (2009). HaCaT keratinocytes in co-culture with Staphylococcus aureus can be protected from bacterial damage by polihexanide. Wound Repair Regen..

[9] Serra R., Grande R., Butrico L., Rossi A., Settimio U.F., Caroleo B., Amato B., Gallelli L., de Franciscis S. (2015). Chronic wound infections: The role of Pseudomonas aeruginosa and Staphylococcus aureus. Expert Rev. Anti-Infect. Ther..

[10] Santajit S., Indrawattana N. (2016). Mechanisms of Antimicrobial Resistance in ESKAPE Pathogens. BioMed Res. Int..

[11] Ngo Q.V., Faass L., Sähr A., Hildebrand D., Eigenbrod T., Heeg K., Nurjadi D. (2022). Inflammatory Response Against Staphylococcus aureus via Intracellular Sensing of Nucleic Acids in Keratinocytes. Front. Immunol..

[12] Roy S., Santra S., Das A., Dixith S., Sinha M., Ghatak S., Ghosh N., Banerjee P., Khanna S., Mathew-Steiner S. (2020). Staphylococcus aureus Biofilm Infection Compromises Wound Healing by Causing Deficiencies in Granulation Tissue Collagen. Ann. Surg..

[13] Wilkinson H.N., Hardman M.J. (2020). Wound healing: Cellular mechanisms and pathological outcomes. Open Biol..

[14] Dubey A.K., Podia M., Priyanka, Raut S., Singh S., Pinnaka A.K., Khatri N. (2021). Insight Into the Beneficial Role of Lactiplantibacillus plantarum Supernatant Against Bacterial Infections, Oxidative Stress, and Wound Healing in A549 Cells and BALB/c Mice. Front. Pharmacol..

[15] Guo S., Dipietro L.A. (2010). Factors affecting wound healing. J. Dent. Res..

[16] Zhang M., Jiang Z., Li D., Jiang D., Wu Y., Ren H., Peng H., Lai Y. (2015). Oral Antibiotic Treatment Induces Skin Microbiota Dysbiosis and Influences Wound Healing. Microb. Ecol..

[17] Lipsky B.A., Hoey C. (2009). Topical antimicrobial therapy for treating chronic wounds. Clin. Infect. Dis..

[18] Han G., Ceilley R. (2017). Chronic Wound Healing: A Review of Current Management and Treatments. Adv. Ther..

[19] Kesavelu D., Jog P. (2023). Current understanding of antibiotic-associated dysbiosis and approaches for its management. Ther. Adv. Infect. Dis..

[20] Ng W.Z.J., van Hasselt J., Aggarwal B., Manoharan A. (2023). Association Between Adult Antibiotic Use, Microbial Dysbiosis and Atopic Conditions—A Systematic Review. J. Asthma Allergy.

[21] Zhao G., Usui M.L., Lippman S.I., James G.A., Stewart P.S., Fleckman P., Olerud J.E. (2013). Biofilms and Inflammation in Chronic Wounds. Adv. Wound Care.

[22] Pinto S., Fumincelli L., Mazzo A., Caldeira S., Martins J.C. (2017). Comfort, well-being and quality of life: Discussion of the differences and similarities among the concepts. Porto Biomed. J..

[23] Brandi J., Cheri S., Manfredi M., Di Carlo C., Vita Vanella V., Federici F., Bombiero E., Bazaj A., Rizzi E., Manna L. (2020). Exploring the wound healing, anti-inflammatory, anti-pathogenic and proteomic effects of lactic acid bacteria on keratinocytes. Sci. Rep..

[24] Mani-López E., Arrioja-Bretón D., López-Malo A. (2022). The impacts of antimicrobial and antifungal activity of cell-free supernatants from lactic acid bacteria in vitro and foods. Compr. Rev. Food Sci. Food Saf..

[25] Aiba Y., Umeda K., Rahman S., Nguyen S.V., Komatsu Y. (2019). Synergistic effect of anti-Helicobacter pylori urease immunoglobulin Y from egg yolk of immunized hens and Lactobacillus johnsonii No.1088 to inhibit the growth of Helicobacter pylori in vitro and in vivo. Vaccine.

[26] Drumond M.M., Tapia-Costa A.P., Neumann E., Nunes Á.C., Barbosa J.W., Kassuha D.E., Mancha-Agresti P. (2023). Cell-free supernatant of probiotic bacteria exerted antibiofilm and antibacterial activities against Pseudomonas aeruginosa: A novel biotic therapy. Front. Pharmacol..

[27] Youssef D.A., Miller C.W., El-Abbassi A.M., Cutchins D.C., Cutchins C., Grant W.B., Peiris A.N. (2011). Antimicrobial implications of vitamin D. Dermato-Endocrinology.

[28] Liu W., Zhang L., Xu H.-J., Li Y., Hu C.-M., Yang J.-Y., Sun M.-Y. (2018). The Anti-Inflammatory Effects of Vitamin D in Tumorigenesis. Int. J. Mol. Sci..

[29] Sassi F., Tamone C., D’Amelio P. (2018). Vitamin D: Nutrient, Hormone, and Immunomodulator. Nutrients.

[30] Thomason J., Rentsch C., Stenehjem E.A., Hidron A.I., Rimland D. (2015). Association between vitamin D deficiency and methicillin-resistant Staphylococcus aureus infection. Infection.

[31] Yokosawa E.B., Arthur A.E., Rentschler K.M., Wolf G.T., Rozek L.S., Mondul A.M. (2018). Vitamin D intake and survival and recurrence in head and neck cancer patients. Laryngoscope.

[32] Wang D., Lin L., Lei K., Zeng J., Luo J., Yin Y., Li Y., Zhang L., Nie X., Zuo D. (2020). Vitamin D3 analogue facilitates epithelial wound healing through promoting epithelial-mesenchymal transition via the Hippo pathway. J. Dermatol. Sci..

[33] Patria F.F., Ceccarini M.R., Codini M., Conte C., Perioli L., Beccari T., Albi E. (2019). A Role for Neutral Sphingomyelinase in Wound Healing Induced by Keratinocyte Proliferation upon 1α, 25-Dihydroxyvitamin D3 Treatment. Int. J. Mol. Sci..

[34] Bikle D.D. (2023). Role of vitamin D and calcium signaling in epidermal wound healing. J. Endocrinol. Investig..

[35] de Carvalho Dias K., Barbugli P.A., de Patto F., Lordello V.B., de Aquino Penteado L., Medeiros A.I., Vergani C.E. (2017). Soluble factors from biofilm of Candida albicans and Staphylococcus aureus promote cell death and inflammatory response. BMC Microbiol..

[36] Iwamoto K., Moriwaki M., Miyake R., Hide M. (2019). Staphylococcus aureus in atopic dermatitis: Strain-specific cell wall proteins and skin immunity. Allergol. Int..

[37] Wang J.W., Hogan P.G., Hunstad D.A., Fritz S.A. (2015). Vitamin D sufficiency and Staphylococcus aureus infection in children. Pediatr. Infect. Dis. J..

[38] Tomic-Canic M., Burgess J.L., O’Neill K.E., Strbo N., Pastar I. (2020). Skin Microbiota and its Interplay with Wound Healing. Am. J. Clin. Dermatol..

[39] Saravanan P., Pooja R., Balachander N., K. K.R.S., S. S., S. R. (2023). Anti-inflammatory and wound healing properties of lactic acid bacteria and its peptides. Folia Microbiol..

[40] Zanetta P., Squarzanti D.F., Sorrentino R., Rolla R., Aluffi Valletti P., Garzaro M., Dell’Era V., Amoruso A., Azzimonti B. (2021). Oral microbiota and vitamin D impact on oropharyngeal squamous cell carcinogenesis: A narrative literature review. Crit. Rev. Microbiol..

[41] Mousa A., Misso M., Teede H., Scragg R., de Courten B. (2016). Effect of vitamin D supplementation on inflammation: Protocol for a systematic review. BMJ Open.

[42] Lee J., Kim S., Kang C.-H. (2022). Immunostimulatory Activity of Lactic Acid Bacteria Cell-Free Supernatants through the Activation of NF-κB and MAPK Signaling Pathways in RAW 264.7 Cells. Microorganisms.

[43] Kasti A.N., Synodinou K.D., Pyrousis I.A., Nikolaki M.D., Triantafyllou K.D. (2021). Probiotics Regulating Inflammation via NLRP3 Inflammasome Modulation: A Potential Therapeutic Approach for COVID-19. Microorganisms.

[44] Alva-Murillo N., Téllez-Pérez A.D., Medina-Estrada I., Álvarez-Aguilar C., Ochoa-Zarzosa A., López-Meza J.E. (2014). Modulation of the inflammatory response of bovine mammary epithelial cells by cholecalciferol (vitamin D) during Staphylococcus aureus internalization. Microb. Pathog..

[45] De Marco S., Sichetti M., Muradyan D., Piccioni M., Traina G., Pagiotti R., Pietrella D. (2018). Probiotic Cell-Free Supernatants Exhibited Anti-Inflammatory and Antioxidant Activity on Human Gut Epithelial Cells and Macrophages Stimulated with LPS. Evid.-Based Complement. Altern. Med..

